# NeatMap - non-clustering heat map alternatives in R

**DOI:** 10.1186/1471-2105-11-45

**Published:** 2010-01-22

**Authors:** Satwik Rajaram, Yoshi Oono

**Affiliations:** 1 Department of Physics, 1110 W. Green Street, University of Illinois at Urbana-Champaign, Urbana, IL 61801-3080, USA; 2 Institute for Genomic Biology, University of Illinois at Urbana-Champaign, Urbana, IL 61801, USA; 3 Current address: Department of Pharmacology, University of Texas Southwestern Medical Center, Dallas, TX 75390, USA

## Abstract

**Background:**

The clustered heat map is the most popular means of visualizing genomic data. It compactly displays a large amount of data in an intuitive format that facilitates the detection of hidden structures and relations in the data. However, it is hampered by its use of cluster analysis which does not always respect the intrinsic relations in the data, often requiring non-standardized reordering of rows/columns to be performed post-clustering. This sometimes leads to uninformative and/or misleading conclusions. Often it is more informative to use dimension-reduction algorithms (such as Principal Component Analysis and Multi-Dimensional Scaling) which respect the topology inherent in the data. Yet, despite their proven utility in the analysis of biological data, they are not as widely used. This is at least partially due to the lack of user-friendly visualization methods with the visceral impact of the heat map.

**Results:**

NeatMap is an R package designed to meet this need. NeatMap offers a variety of novel plots (in 2 and 3 dimensions) to be used in conjunction with these dimension-reduction techniques. Like the heat map, but unlike traditional displays of such results, it allows the entire dataset to be displayed while visualizing relations between elements. It also allows superimposition of cluster analysis results for mutual validation. NeatMap is shown to be more informative than the traditional heat map with the help of two well-known microarray datasets.

**Conclusions:**

NeatMap thus preserves many of the strengths of the clustered heat map while addressing some of its deficiencies. It is hoped that NeatMap will spur the adoption of non-clustering dimension-reduction algorithms.

## Background

With the advent of high-throughput experiments, whole genome measurements across multiple conditions have become common. Human pattern recognition is still unmatched by computers, making it advantageous to visualize this data. Over the past decade, the clustered heat map has become by far the most popular visualization technique. It has been used in thousands of publications spanning a multitude of organisms and a variety of data types [1-3]; it has even been dubbed [4] a "post genomic visual icon." There are good reasons for the clustered heat map's popularity. It provides a compact, easy to grasp, depiction of a large amount of data across two variables (*e.g.*, gene and sample) with large contiguous bands of similar colors that encourage the formulation of more general hypotheses between these variables. Still, the clustered heat map has some glaring flaws. As its name suggests, the rows and columns are ordered using hierarchical clustering algorithms (while there are other clustering schemes [5], they are typically not used to construct heatmaps, so here, clustering should be understood to refer to hierarchical clustering). Distances in a clustering result are measured along the tree branches and not by the proximity in branch tip ordering. While these measures are related (especially for very similar elements), they could be very different [6]. Additionally, during clustering, when objects are assigned to different clusters, further analysis essentially involves these clusters as a whole, and the relationship between the elements themselves is lost (see analysis of human gene atlas in Results). Consequently, clustering does not provide any natural ordering; the rows and columns may be reordered arbitrarily by 'swinging' the arms of the tree at each bifurcation yet preserving the tree structure. The ordering produced by clustering thus does not respect the intrinsic topology (if any) of the data, making it a poor choice for use in a heat map. This is why 'swinging' based reordering using an independent method is often required, post-clustering, to capture the structure of the data. There are two problems with this reordering. Firstly, unlike the clustering schemes, the reordering algorithms, while complex enough to warrant dedicated software packages, are often not elaborated upon or even stated. This reduces the reproducibility of the result. More seriously, this procedure could potentially place (deliberately or otherwise) objects that are distant along the tree in close proximity in the row/column order. Heat maps are commonly read in this order rather than by their dendrogram structure (if this were not the case, such reordering schemes would not be needed). Effectively a spurious pattern could be created, leading to incorrect results (*e.g.*, see clustered heat map for Spellman data in Results).

So far we assumed the clustering results themselves were meaningful. Indeed, when the underlying data is tree-like, or at least some clustering/grouping tendency is present, cluster analysis+reordering performs well. However, this is not always the case. As group separation becomes fuzzier, other data-reduction schemes often outperform cluster analysis. Usually, it is considered good practice to test for clustering tendency before performing clustering or to perform bootstrap-like methods to estimate cluster quality post-clustering [7]. Unfortunately, this kind of information is not typically provided in a heat map. Thus validation is only by visual inspection of the color patterns, and this may be misleading.

Biological data often has a low dimensional structure that may be visualized as a spatial pattern, so direct use of a suitable dimension-reducing algorithm could, in many cases, be more natural and better characterize the data than the current combination of structure destroying clustering + restoring algorithm. There are many such algorithms whose utility in the analysis of biological data has been demonstrated [8,9]. Multiple packages in R [10], and otherwise, implement them. Despite this, we believe their use has been limited, at least partially, by the lack of associated visualization methods with the visceral impact of the clustered heat map.

Here we present an R package called NeatMap to meet this need while addressing some of the deficiencies of the clustered heat map. It consists of novel plot-types in two and three dimensions intended to be used in conjunction with any dimension-reduction scheme capable of embedding results in low dimensional Euclidean space (*e.g.*, Principal Component Analysis (PCA) and Multi-Dimensional Scaling (MDS)). This places weaker constraints on the data than does (hierarchical) cluster analysis, which requires the data to exist in a tree space. Like the heat map, and unlike typical visualization schemes for these methods, NeatMap displays the entire dataset underlying the result. It also has provisions to superimpose the cluster analysis results, for mutual validation. This feature is not commonly implemented in software packages, and our implementation is more informative about individual points than existing implementations [11]. Also note that unlike the clustered heat map, the layout of the plot is almost entirely determined by the output of the dimension-reduction scheme, thereby respecting the intrinsic structure in the data more than a clustering based reordering would.

There are a number of alternatives to hierarchical clustering (see, for example, the R package seriation [12]), designed specifically to produce an ordering that reflects the relative relations between elements. NeatMap is a visualization method, and in general it is not intended to compete with these (in fact they can easily be used in conjunction). However, some of these techniques involve ordering by the first component of PCA/MDS. Unless, this component captures most of the relevant information, NeatMap, which uses 2D embeddings, is likely to better utilize the dimensional reduction results. On the other hand, we do not consider alternate clustering algorithms such as *k*-means clustering [13], tight clustering [14] and various model based clustering algorithms [15-17]. Although these avoid some of the problems faced by hierarchical clustering as outlined above, and have been shown to perform better [5], they typically just assign (or give probabilities of assigning) objects to clusters. No relations among objects within a cluster are provided, and typically the relations among clusters is not used either. Thus, they do not naturally support the construction of heatmap like plots. Self Organizing Maps (SOM) [18] used with a small number of nodes/clusters face a similar problem. However, as the number of clusters increases, they essentially involve mapping objects onto points in a low dimensional space much like multidimensional scaling. In this case, it should be possible to use SOMs in conjunction with NeatMap, although we have not considered it in this paper. Methods such as model based clustering do not presently have associated visualization methods, but if their results could somehow be mapped onto points in Euclidean space, they too could be visualized with the help of NeatMap. Note that NeatMap analyzes the rows and columns of the gene expression matrix separately, and is therefore not intended to visualize bi-clustering results.

## Implementation

The general class of data considered involves factors (*e.g.*, genes) being measured across multiple conditions (*e.g.*, samples, times, tissues, etc.). For each factor, these measurements will be referred to as its profile. It is assumed here that some dimension-reduction scheme, (*e.g.*, PCA) has been used to depict the relationship between factors by embedding them into a 2D Euclidean space. The plots described here allow us to visualize these relationships, while simultaneously showing the profiles underlying them. NeatMap may be used to visualize the results produced by any appropriate dimensional reduction scheme of the user's choice. For the case when the user does not already have a dimensionally reduced result, NeatMap can itself invoke and then visualize (the results of) one of two dimensional reduction methods:

1. Principal Component Analysis (PCA) [19] produces a low dimensional representation of the data using the linear combinations of variables that capture the maximum amount of variance. Being a linear scheme, it is very fast, although this may sometimes be at the expense of quality of result.

2. non-Metric Multi-Dimensional Scaling (nMDS) [20,21] is a dimensional reduction scheme that attempts to represent factors as points in a low dimensional Euclidean space such that the (relations among) distances between the points in the low dimensional space are consistent with those in the original data. nMDS is a non-linear scheme that is typically found to outperform PCA, but is slower for large data sets.

**Figure 1:**
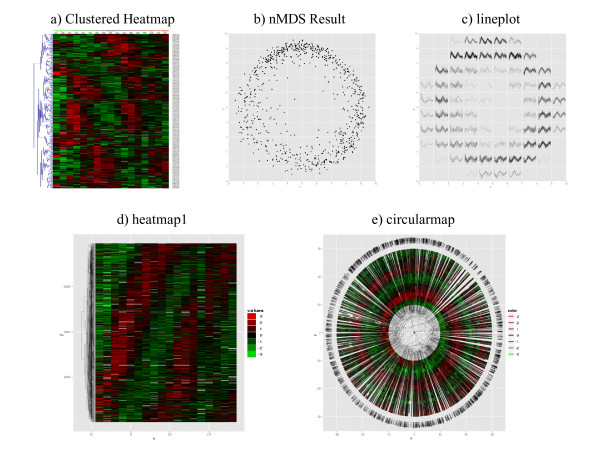
**Different ways of representing the cyclic genes for the alpha experiment in Spellman *et al. ***[26]. (a) is the standard heat map using average-linkage hierarchical clustering in MeV, shown here for comparison. (b) is the result of 2D nMDS. The profiles for all the genes in each grid cell in (b) are shown using **lineplot **in the corresponding grid cell in (c). (d) shows **heatmap1 **in which the angular positions of genes in (b) is used to reorder the rows in (a). (e) is **circularmap **using the angular positions of points in (b).

**Figure 2:**
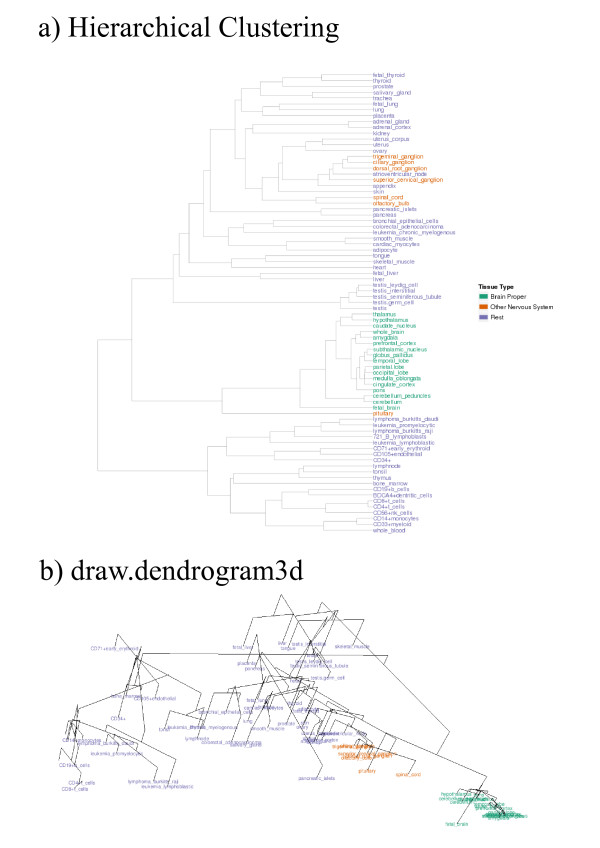
**Representations of the tissue relations in the human gene atlas data**: (a) is the average-linkage hierarchical clustering (using Pearson correlation) result applied to the tissues; (b) shows the superimposition of the clustering result on a 2D nMDS embedding of tissues using **draw.dendrogram3d**.

**Figure 3:**
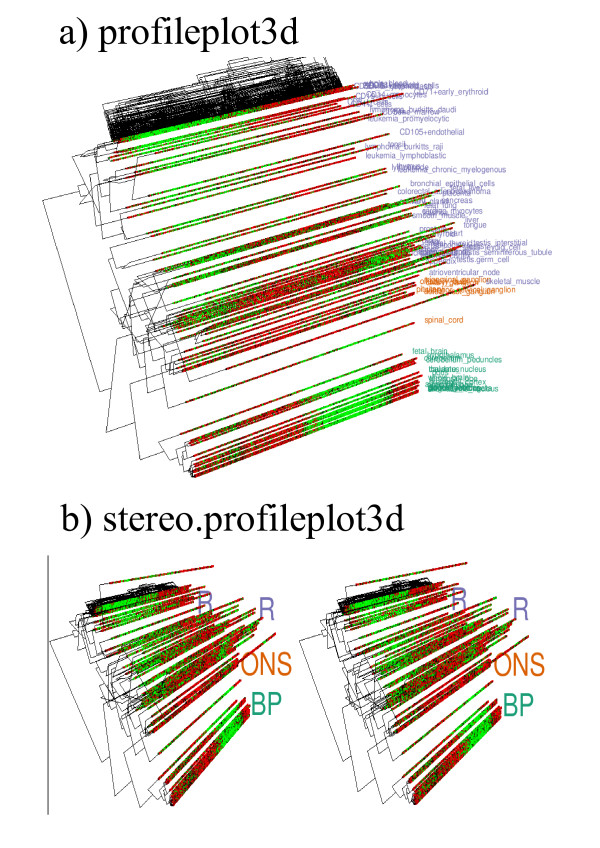
**Representations of the human gene atlas data**: (a) shows the expression profiles underlying figure 2(b) using **profileplot3d**. The different groups of tissues are marked with labels of differing colors. (b) is a stereo plot of the same result created using **stereo.profileplot3d**. (BP = Brain Proper, ONS = Other Nervous System and R = Rest)

The utility of both methods in the analysis of gene-expression data has previously been shown [8,9,22]. Based on the performance differences between nMDS and PCA, we suggest that if less than 3000 points are being used, nMDS should be used, while PCA is better for larger sets (at least on an ordinary laptop computer). nMDS was used as the dimension-reduction scheme for the demonstrations in this paper, because, generally speaking, the embedding produced by nMDS is more informative than the corresponding PCA result (results for larger data sets embedded using PCA can be seen in Additional File 1). An R implementation of nMDS is included for convenience in the package. There are multiple plots in this package, each emphasizing different aspects of the factor-condition relationship:

1. **heatmap1**: This is the traditional heat map, except a dimension-reduction scheme other than clustering (for examples see [12]) may be used for ordering of rows and/or columns. NeatMap itself provides a novel way to do this from a 2D embedding method: normalize the data, or use an amplitude neutral distance measure such as the Pearson correlation. Then, the embedded result produced by PCA, nMDS, etc., is often annular and can be parameterized, approximately, by a single variable, *viz.*, the angular position (figure 1d). This is a better option than using the ordering based on a single component. The standard cluster dendrogram may be superimposed on the heat map for mutual validation.

2. **circularmap**: Similar to **heatmap1 **except the arrangement is circular (figure 1e) rather than linear to emphasize the periodicity of the angular positions obtained as above (or using other methods [23] that produce annular results). It is easy to make comparisons across conditions and factors. The factor clustering result may be superimposed on this plot.

3. **lineplot**: The 2D dimensionally-reduced factor relationship result is gridded, and the profiles of all the factors within each grid cell are displayed together as line graphs (figure 1c). This provides a global understanding of the nature of the data and its embedding. However, individual factors are harder to pick out, and comparison across conditions is more difficult.

4. **draw.dendrogram3d**: Cluster validation of the 2D embedding result for factors (figure 2b) in a 3D environment. The clustering result for both factors and conditions may be superimposed on **profileplot3d**.

5. **profileplot3d**: Addresses the inability of **heatmap1 **and **circularmap **to depict radial information by visualizing the profiles in a 3rd dimension using a rotatable 3D environment (figure 3a).

6. **stereo.profileplot3d**: A stereo plot where two versions of the same **profileplot3d **result are shown as viewed from slightly different perspectives to produce the impression of a true 3D view (figure 3b). The plot may be rotated dynamically to provide different views. This plot should also be useful for producing 3D plots for publications where rotation is not possible.

The functions above are dimension-reduction method neutral; dimensionally-reduced results provided by the user are plotted. Convenience wrapper functions **make.heatmap1**, **make.circularmap**, **make.profileplot3d **and **make.stereo.profileplot3d **are also provided. They take just the raw data as input, perform dimension-reduction using either nMDS or PCA, and finally produce the appropriate plots. All 2D plots were implemented by using **ggplot2 **[24] and 3D plots using **rgl **[25]. These libraries have numerous functions for additional customization and modification of the plots produced by NeatMap.

## Results and Discussion

The utility of the plots described above are demonstrated with the aid of two different microarray-based datasets. The 2D plots are illustrated with the help of the Spellman *et al. *[26] dataset identifying cell cycle related genes in yeast, while microarray data from the human gene atlas study [27], profiling gene expression across multiple tissues, is used for the 3D plots.

### 2D plots

Spellman *et al. *[26] produced genome-wide time course profiles in yeast using micro-arrays under different synchronization methods. Fourier analysis was then used to identify 800 genes, with the correct periodicity, as cell cycle related. We consider only these 800 cell cycle related genes and study their profiles under *alpha *synchronization. For an example with a larger number of points without such periodicity see Additional File 1. Since a natural time ordering of the measurements exists, we are only interested in the relationship between genes.

For comparison to the plots produced by **NeatMap **we used the Multiexperiment Viewer (MeV) software to generate the standard clustered heat map for this data (figure 1a). Average linkage hierarchical clustering of the Pearson correlation, followed by MeV's function for optimal reordering of genes were used. Although the periodicity of these genes is clear, and locally good groupings are seen, the pattern as a whole appears quite jagged. This is because a cluster like topology was forced on an essentially continuous distribution. Closely related groups of genes are correctly clustered together but the global relations between genes in different clusters (which is essential for complete ordering) are lost. Figure 1b shows the result produced by a 2D embedding of the gene profiles using nMDS, again with the Pearson correlation. A clear continuous ring like pattern emerges naturally. (PCA, with normalized profiles, shows a similar result although the ring structure is more diffuse; see Additional File 2).

Such a ring-like structure is very common when an amplitude-normalized distance measure such as the Pearson correlation is used. In this situation, it is natural to parameterize the position of a gene by a single angle. This is what **heatmap1 **does. For each gene, its angular position in the nMDS result (figure 1b), with respect to its center of mass, is determined, and the profiles are placed (figure 1d) in a standard heat map ordered according to this angle. The periodic nature of the profiles is now clear, and it is evident that points are arranged by time of up-regulation; essentially the cell cycle phase in which the gene is expressed. While in this case the angular co-ordinate was interpretable as the cell cycle phase, this method works even with non-periodic data when such interpretation is not the possible (see, for example, Additional File 1). Note that **heatmap1 **also accepts orderings produced by other methods. The R package seriation [12] offers a variety of these, and **heatmap1 **plots using them for the Spellman data set are available as Additional File 3. In general, the NeatMap ordering is superior, except for the case of Rank Two Ellipse [23]. This method, like NeatMap, uses angular ordering based on normalized profiles (the correlation matrix itself in this case). **heatmap1 **also allows the superimposition of clustering results. Evidently, the local arrangements in nMDS and clustering are consistent. Large scale rearrangement, produced by incorrect 'swinging', however, makes the clustered heat map result seem poor.

There are some long lines in the gene clustering result in figure 1c spanning the entire length of the heat map. This is a consequence of the periodicity of the angular variable, which results in the two opposite ends of the heat map being almost identical. To avoid artifacts from this periodicity, one may use **circularmap **(figure 1e). The ordering of profiles is identical to **heatmap1**, except they are placed along a circle according to their angular positions in figure 1b. One additional advantage of this format is that the non-uniformity in the phase distribution stands out more clearly. It is much harder to gain this type of information from a traditional heat map display.

Figure 1c shows the **lineplot **based on the nMDS result in figure 1b. As explained earlier, each cell in the grid in figure 1c shows the time course profiles of all the genes in the corresponding cell in figure 1b. The sinusoidal nature of the profiles is much clearer in this plot. It also emerges that the radial coordinate in this case is a measure of 'cyclicity', with the genes close to the centre being less cyclic.

Thus, **lineplot **emphasizes the overall nature and change in profiles with position. However, compared to **heatmap1 **and **circularmap**, comparison of expression at a fixed time across genes is more difficult. It is also more difficult to quickly look up a specific gene. On the other hand, **heatmap1 **and **circularmap **are intended for essentially one dimensional results. To deal with the more general case we must use 3D rotatable plots.

Assuming the profiles are stored in matrix form in alpha.profiles, the code to produce figure 1c, d, and 1e (except for specific graphics options) is:

pos.nMDS<-nMDS(alpha.profiles)$x;# Perform nMDS embedding

lineplot(pos.nMDS,alpha.profiles,normalize=T); #1c

make.heatmap1(alpha.profiles,row.normalize=T); #1d

make.circularmap(alpha.profiles); #1e

To use PCA instead of nMDS, a single parameter specifying this would need to be added to each of these plots.

### 3D plots

We illustrate the 3D plots using the gene atlas dataset. Su *et al. *[27] used microarrays to analyze the expression profiles of genes in a variety of tissues in both humans and mouse. There is no natural ordering of the genes or tissues, but the relationships between tissues are more easily understood. We therefore primarily focus on these.

Since, in the present context, we are not interested in cross-species comparison, for this demonstration only human data was used (mouse gives similar results). The 1000 genes on the HG-U133A array showing largest variance across the 79 tissues were analyzed. Functionally, there are broadly 3 groups of tissues: those from the brain proper, some nervous system related tissues, and those from other parts of the body. The result of applying hierarchical clustering (average-linkage) using the Pearson correlation to the tissues is shown in figure 2a. Three distinct clusters are seen, one of which is composed solely of brain tissues. However, the nervous tissues are mixed with the other non-brain tissues in the second cluster and no relation to the brain can be gleaned from the leaf order or distance along the tree.

A 2D embedding of the same data using nMDS with Pearson correlation was also performed. The cluster analysis result was superimposed on the 2D nMDS result in a rotatable 3D environment using **draw.dendrogram3d **(figure 2b). The same three clusters are present, and there is broad agreement between the clustering and nMDS results. Unlike the clustering result, however, the relationship between the brain and nervous system tissues is much clearer. The nervous system genes are also quite similar to the central cluster of tissues in figure 2b. Apparently, cluster analysis assigns them to this cluster, and in doing so their relationship to the proper brain tissues is lost.

The profiles underlying the nMDS result may be displayed in a rotatable 3D environment by using **profileplot3d**. Figure 3a shows this with the cluster analysis results for genes and tissues superimposed on it. The genes were ordered according to their angular positions in a ring-like nMDS embedding by making use of the Pearson correlation, much like **heatmap1**. The separation between the three groups of tissues can be seen as before. However, **profileplot3d **makes it clear that there are different set of genes up-regulated in these groups. The same result can be viewed as a rotatable stereo plot using **stereo.profileplot3d **(figure 3b). This type of plot could be useful for publications and other environments where dynamic rotations are not possible.

Assuming the data is stored in matrix form (with genes along the rows and tissues along columns) in atlas.profiles, the cluster analysis result for tissues in atlas.cluster, and the three groups are color coded in atlas.group.colors the code to produce the plots in figure. 2 and 3 are:

atlas.nMDS<-nMDS(profiles)$x;

draw.dendrogram3d(atlas.nMDS,atlas.cluster,labels=colnames(atlas.profiles),

label.colors=atlas.group.colors);

make.profileplot3d(atlas.profiles,column.method="nMDS",

labels=colnames(atlas.profiles),label.colors=atlas.group.colors);

make.stereo.profileplot3d(atlas.profiles,column.method="nMDS",

labels=colnames(atlas.profiles),label.colors=atlas.group.colors);

## Conclusions

The clustered heat map, an immensely popular means to visualize large amounts of data, is encumbered by its dependence on cluster analysis. Many alternative dimension-reduction schemes have the potential to do better, but have so far lacked effective means to visualize whole datasets in the way the heat map can. **NeatMap **is an R package that addresses this need. Using the well-known Spellman yeast cell-cycle and human gene atlas microarray datasets, we have shown that a dimension-reduction method (nMDS was used in this paper for illustration) in conjunction with **NeatMap **is more informative than the clustered heat map. It is hoped that this package will increase the popularity of these methods and spur the development of novel visualization schemes.

## Availability and requirements

**Project name**: NeatMap

**Project home page**: http://cran.r-project.org/web/packages/NeatMap/index.html

**Operating system(s)**: Platform independent

**Programming language**: R

**Other requirements**: R, R packages(ggplot2 and rgl)

**License**: GPL-3

## Authors' contributions

SR designed, created the software and drafted the manuscript. YO conceived of the project, helped in the design of the software and drafting of the manuscript. Both authors read and approved the final manuscript.

## Supplementary Material

Additional file 1 **Analysis of the gene atlas data using PCA and NeatMap**. Unlike in the analysis of the gene atlas data in the main text, where the expression profiles of only 1000 ESTs were considered, here we analyzed all 13,034 ESTs. The tissue and gene expression profiles were both normalized to zero mean and unit variance. Both the gene and tissue profiles were analyzed using PCA and were represented using the first two principal components. The gene expressions results lay in a circular region and were therefore parametrized/ordered by their angular positions. The tissue result was more skewed and we therefore ordered tissues according to their first principal component. (a) shows the result using **heatmap1 **with the rows (genes) ordered by the angular position of the 2D PCA embedding and the columns (tissues) ordered according their first principal component. (b) shows the **circularmap **result using the angular position and tissue ordering as described above. Both plots clearly place similar genes and tissue close to each other, although there is no simple interpretation of the angular variable as in the case of cell cycle data. Click here for file

Additional file 2 **Spellman *et al. ***[26]**data analyzed using PCA and NeatMap**. The NeatMap plots in figure 1 produced using PCA instead of nMDS. Spellman *et al. *data using *alpha *synchronization was visualized using PCA and NeatMap. The profiles were normalized to have zero mean and unit variance, and all profiles with missing data were discarded (a) is the standard PCA result, (b), (c) and (d) show the **lineplot**, **heatmap1 **and **circularmap **functions respectively applied to (a). Click here for file

Additional file 3 **heatmap1 for the Spellman data **[26]**using different ordering schemes in the R package seriation **[12]. **heatmap1 **may be used in conjunction with orderings produced using external algorithms. The R package seriate [12] contains a number of these. **heatmap1 **using the Spellman data [26] and different ordering schemes using seriate are shown in the figure. a) uses the Travelling Salesman Algorithm, b) orders rows according to the first component of the PCA embedding of the rows, c) is ordering according to elliptic ordering method proposed by Chen [23], d) by the method proposed by Gruvaeus and Wainer, e) by the 1st component of the MDS embedding of rows, f) by the Optimal Leaf Ordering algorithm. Click here for file
