# Supplementary figures and images for: NeatMap - non-clustering heat map alternatives in R
Source: BMC Bioinformatics. 2010 Jan 22;11:45. doi: 10.1186/1471-2105-11-45 (PMC3098076; doi:10.1186/1471-2105-11-45)

a) heatmap1

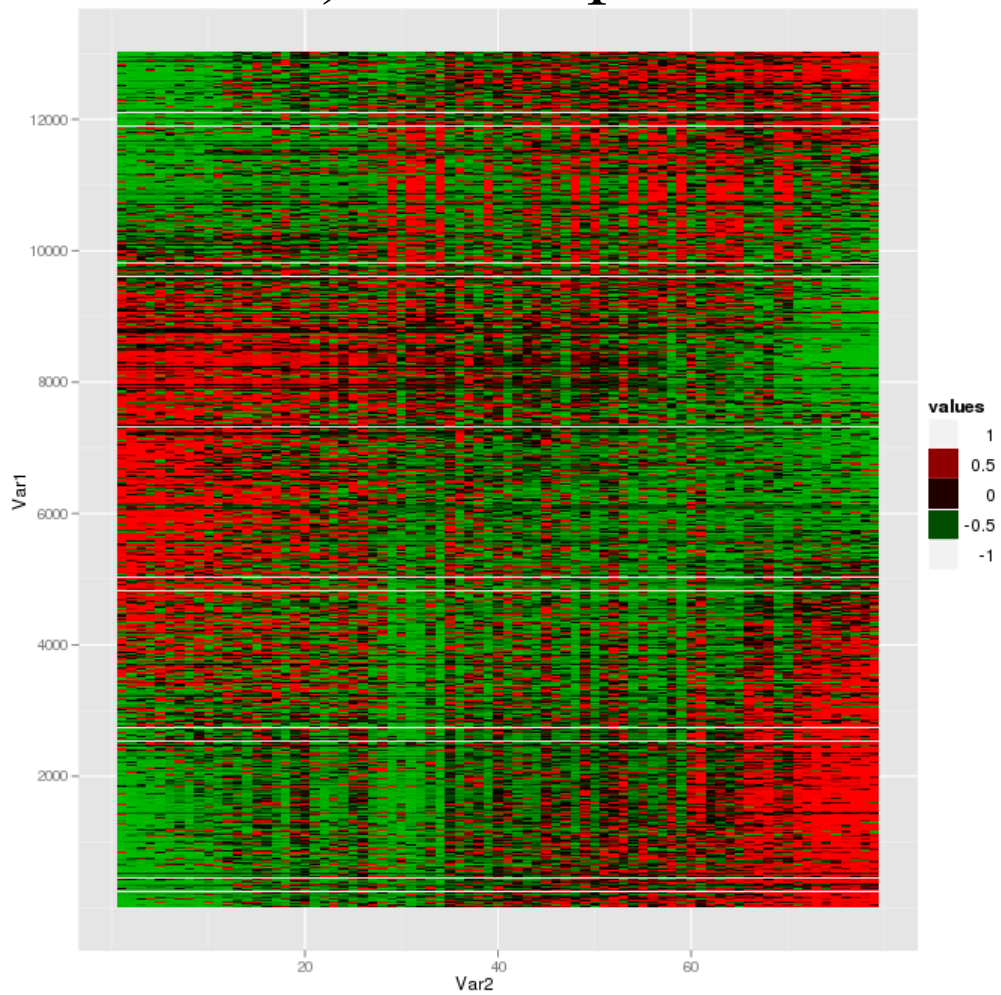

b) circularmap

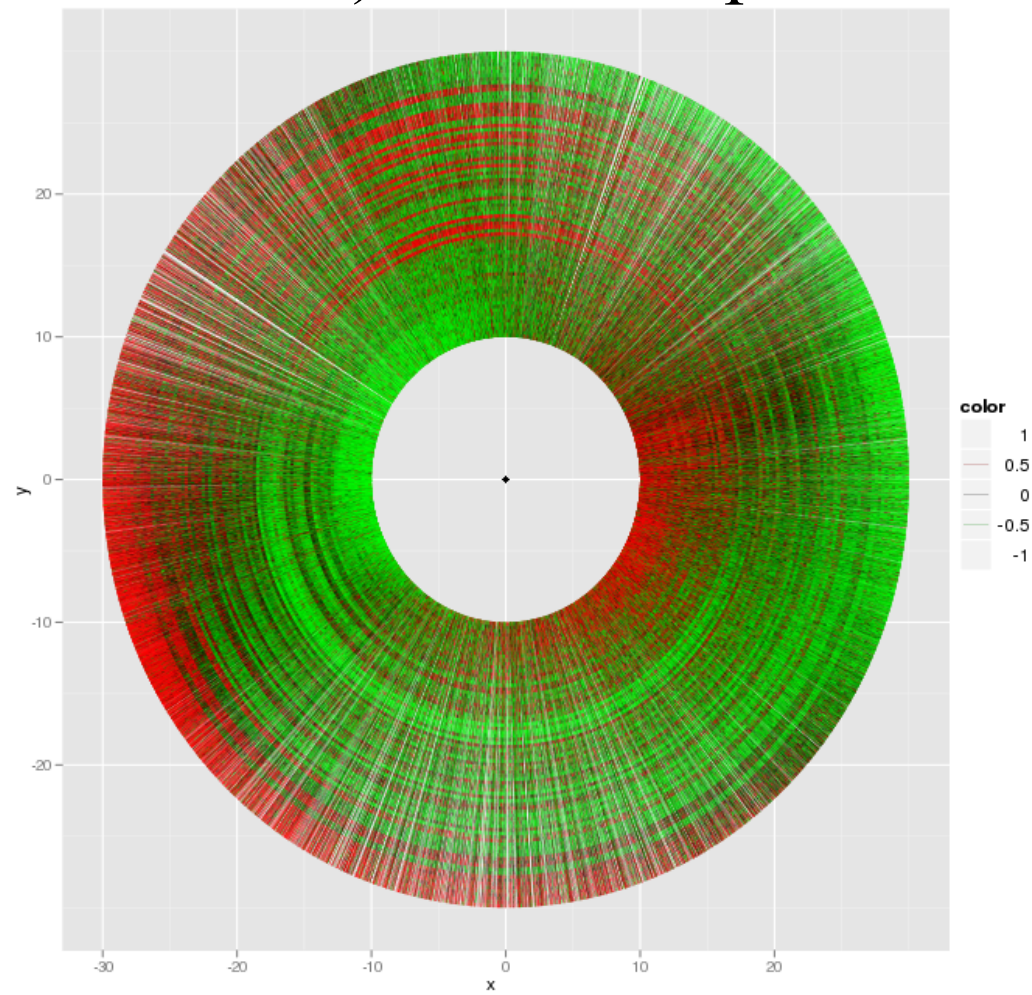

Supplement: Additional file 1 — Analysis of the gene atlas data using PCA and NeatMap. Unlike in the analysis of the gene atlas data in the main text, where the expression profiles of only 1000 ESTs were considered, here we analyzed all 13,034 ESTs. The tissue and gene expression profiles were both normalized to zero mean and unit variance. Both the gene and tissue profiles were analyzed using PCA and were represented using the first two principal components. The gene expressions results lay in a circular region and were therefore parametrized/ordered by their angular positions. The tissue result was more skewed and we therefore ordered tissues according to their first principal component. (a) shows the result using heatmap1 with the rows (genes) ordered by the angular position of the 2D PCA embedding and the columns (tissues) ordered according their first principal component. (b) shows the circularmap result using the angular position and tissue ordering as described above. Both plots clearly place similar genes and tissue close to each other, although there is no simple interpretation of the angular variable as in the case of cell cycle data. [file 1471-2105-11-45-S1.PDF]

a) PCA Result

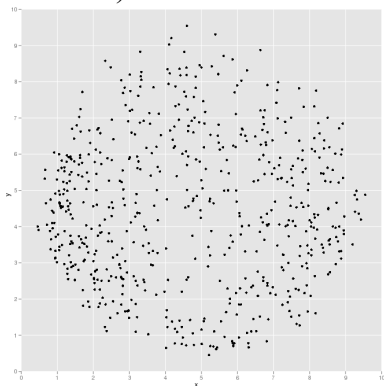

b) lineplot

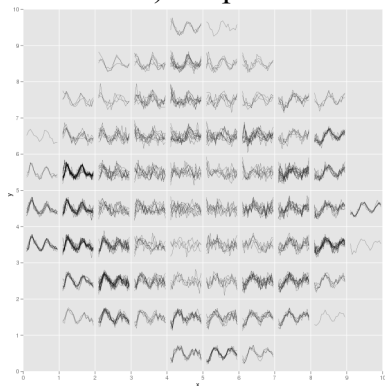

c) heatmap1

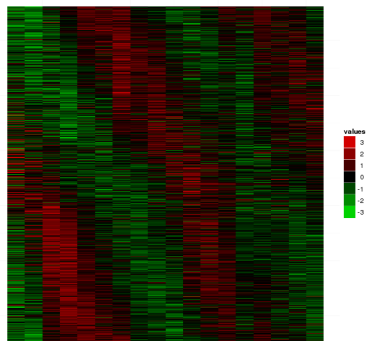

d) circularmap

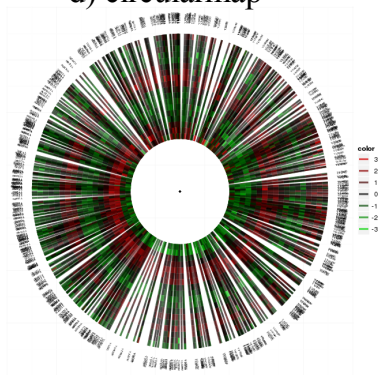

Supplement: Additional file 2 — Spellman et al. [26]data analyzed using PCA and NeatMap. The NeatMap plots in figure 1 produced using PCA instead of nMDS. Spellman et al. data using alpha synchronization was visualized using PCA and NeatMap. The profiles were normalized to have zero mean and unit variance, and all profiles with missing data were discarded (a) is the standard PCA result, (b), (c) and (d) show the lineplot, heatmap1 and circularmap functions respectively applied to (a). [file 1471-2105-11-45-S2.PDF]
