# Supplementary material for: NeatMap - non-clustering heat map alternatives in R
Source: BMC Bioinformatics. 2010 Jan 22;11:45. doi: 10.1186/1471-2105-11-45 (PMC3098076; doi:10.1186/1471-2105-11-45)
Supplement: Additional file 3 — heatmap1 for the Spellman data [26]using different ordering schemes in the R package seriation [12]. heatmap1 may be used in conjunction with orderings produced using external algorithms. The R package seriate [12] contains a number of these. heatmap1 using the Spellman data [26] and different ordering schemes using seriate are shown in the figure. a) uses the Travelling Salesman Algorithm, b) orders rows according to the first component of the PCA embedding of the rows, c) is ordering according to elliptic ordering method proposed by Chen [23], d) by the method proposed by Gruvaeus and Wainer, e) by the 1st component of the MDS embedding of rows, f) by the Optimal Leaf Ordering algorithm. [file 1471-2105-11-45-S3.PDF]

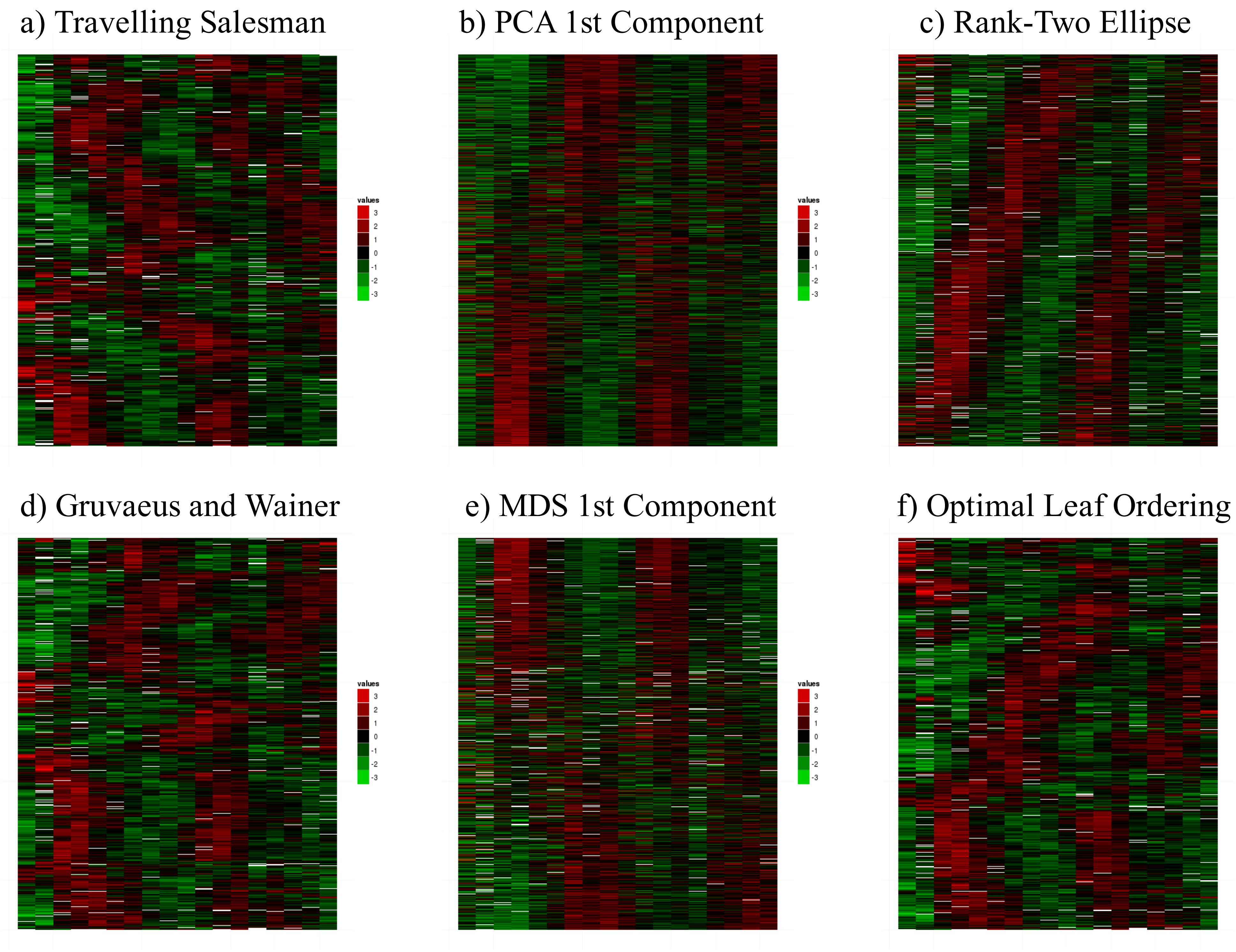

a) Travelling Salesman
b) PCA 1st Component
c) Rank-Two Ellipse
d) Gruvaeus and Wainer
e) MDS 1st Component
f) Optimal Leaf Ordering
values
3
2
1
0
-1
-2
-3
